# Regioselectively Carboxylated Cellulose Nanofibril Models from Dissolving Pulp: C6 via TEMPO Oxidation and C2,C3 via Periodate–Chlorite Oxidation

**DOI:** 10.3390/nano14050479

**Published:** 2024-03-06

**Authors:** Mengzhe Guo, James D. Ede, Christie M. Sayes, Jo Anne Shatkin, Nicole Stark, You-Lo Hsieh

**Affiliations:** 1 Chemical Engineering, University of California at Davis, Davis, CA 95616, USA; mzguo@ucdavis.edu; 2 Vireo Advisors, LLC, P.O. Box 51368, Boston, MA 02130, USA; jede@vireoadvisors.com (J.D.E.); jashatkin@vireoadvisors.com (J.A.S.); 3 Environmental Science, Baylor University, Waco, TX 76798, USA; christie_sayes@baylor.edu; 4 USDA Forest Service, Forest Products Laboratory, Madison, WI 53726, USA; nicole.stark@usda.gov; 5 Biological and Agricultural Engineering, Chemical Engineering, University of California at Davis, Davis, CA 95616, USA

**Keywords:** cellulose nanofibril, TEMPO, periodate–chlorite oxidation, C2,C3 regioselective, dissolving pulp

## Abstract

Regioselective C6 and C2,C3 carboxylated cellulose nanofibrils (CNFs) have been robustly generated from dissolving pulp, a readily available source of unmodified cellulose, via stoichiometrically optimized 2,2,6,6-tetramethylpyperidine-1-oxyl (TEMPO)-mediated and sequential sodium periodate-sodium chlorite (PC) oxidation coupled with high-speed blending. Both regioselectively optimized carboxylated CNF series possess the widest ranges of comparable charges (0.72–1.48 mmol/g for T-CNFs vs. 0.72–1.10 mmol/g for PC-CNFs), but similar ranges of thickness (1.3–2.4 nm for T-CNF, 1.8–2.7 nm PC-CNF), widths (4.6–6.6 nm T-CNF, 5.5–5.9 nm PC-CNF), and lengths (254–481 nm T-CNF, 247–442 nm PC-CNF). TEMPO-mediated oxidation is milder and one-pot, thus more time and process efficient, whereas the sequential periodate–chlorite oxidation produces C2,C3 dialdehyde intermediates that are amenable to further chemical functionalization or post-reactions. These two well-characterized regioselectively carboxylated CNF series represent coherent cellulose nanomaterial models from a single woody source and have served as references for their safety study toward the development of a safer-by-design substance evaluation tool.

## 1. Introduction

Nanocelluloses, the crystalline domains of native cellulose, have been isolated from the cellulose of various sources most commonly by separating chains and/or overcoming the hydrogen bonds in the less ordered regions via mechanical forces [1,2], removing the less crystalline chains via acid [3,4,5] or biochemical enzymatic [6] hydrolysis, reacting the hydroxyls via chemical reactions, such as 2,2,6,6-tetramethylpyperidine-1-oxyl (TEMPO) mediated oxidation [7,8] or a combination of chemical and mechanical means. In the case of acid hydrolysis, the amorphous chains are dissolved leaving the rod-like cellulose nanocrystals (CNCs), typically highly crystalline but in lower yields as expected from removing the less ordered chain segments. On the other hand, shear-force processes separate semicrystalline cellulose into highly branched micro-scaled microfibrils with few cellulose nanofibrils (CNFs) of varying lengths and widths, retaining nearly most, if not all of the original cellulose and thus gives high yields but as highly heterogeneous mixtures. With chemical derivatization, the inter-molecular and inter-fibrillar hydrogen bonds in the amorphous chains are broken to allow fibrillation into more uniform CNFs while the newly induced surface charges also facilitate their aqueous dispersions. The chemical pretreatment of cellulose can significantly reduce the shear energy required and improve the uniformity and yield of the CNFs produced.

We have previously generated nanocelluloses from rice straw cellulose, i.e., CNCs from sulfuric acid hydrolysis [9], CNFs via high-speed blending [9], aqueous counter collision (ACC) [10], and TEMPO-oxidation [9]. As expected, the CNCs from sulfuric acid hydrolysis were highly crystalline but in low yields up to 16.9%. In contrast, the CNFs from shear force processes alone, such as high-speed blending and ACC, were highly branched and heterogeneous in sizes and had yields up to 12% and over 90%, respectively. TEMPO-oxidation alone produced finer CNFs, but also a low yield of 19.7%. Carboxylation, either TEMPO [11] or sequential sodium periodate-sodium chlorite (PC) [12] oxidation, followed by high-speed blending, produced highly uniform CNFs in 97% yields, nearly a full conversion. The same coupled TEMPO-oxidation and blending approach has also been demonstrated to be highly efficient in producing over 92% and 95% CNFs from cellulose isolated from the almond shell [13] and hull [14], respectively.

TEMPO-mediated oxidation is known to regioselectively carboxylate the C6 hydroxyls whereas sodium periodate oxidation breaks the C2-C3 bonds in the anhydroglycose ring to form dialdehydes that can be subsequently sodium chlorite oxidized into C2,C3 dicarboxylates. While they differ in their reaction mechanisms, both TEMPO and PC oxidation reactions are diffusion-controlled and generally occur on cellulose chains in the amorphous regions and in the crystalline-amorphous interfaces. The TEMPO-mediated oxidation of woody biomass has been among the most studied pretreatment for producing nanocelluloses as opposed to the less-reported two-step PC oxidation of hardwood pulp [15,16,17] and soft dissolving pulp [18]. With blending, CNFs in 10–20 nm widths have been reported in yields up to 50% [15] while thinner 2.6–6 nm wide CNFs could be produced in higher 84–100% yields but with an excess of both PC oxidants and aided by microfluidization [16] or high-pressure homogenization [18]. Applications of both TEMPO and periodate oxidations have only been reported on microcrystalline cellulose [19]. Since microcrystalline cellulose is already hydrolyzed (commonly by hydrochloric acid) and mechanically refined from pulp or other sources of cellulose, these oxidations are expected to affect the surfaces of 15–30 μm sized microcrystalline cellulose particles; therefore, they do not provide insight into how these oxidants access and react with the C6 vs. C2 and C3 hydroxl groups in native or chemically unmodified cellulose.

Aqueous TEMPO and PC oxidation reactions are heterogeneous and diffusion-dependent, but these reactions on macro-scaled cellulose have not been studied in parallel, nor systematically elucidated in the context of producing nanocelluloses. Furthermore, how the regioselectivity of these reactions influences the level of oxidation as well as how the regiospecific carboxylation affects the generation of nanocellulose from a specific native cellulose source, and their characteristics have not been articulated or distinguished. 

This study was therefore designed to synthesize CNFs systematically from one mass-produced and well-purified cellulose feedstock, i.e., dissolving soft wood pulp, through a stoichiometrically controlled oxidation via TEMPO and periodate–chlorite oxidation. The goals were to optimize the respective regioselective carboxylation, to discern their reaction efficiency, and to detail the reaction parameters to the structure–property relationships of C6 TEMPO-oxidized T-CNFs and C2,C3 periodate-chlorite oxidized PC-CNFs. This work would contribute to the further understanding of these heterogeneous reactions on cellulose, nature’s most abundant polymer, as well as two comparable series of model CNFs. Furthermore, this work was undertaken as part of a study to provide rationally synthesized and well-characterized CNFs to represent C6 vs. C2,C3 carboxylated and C6-sulfated [20] functionalization toward the development of a toolbox of safer-by-design substance evaluation tools linking the biological behavior of CNFs to their surface chemistries, regioselectivity, charges, and dimensions [21].

## 2. Experimental

### 2.1. Materials 

Hydrochloric acid (HCl, 1N, Certified, Fisher Scientific, Waltham, MA, USA), sodium hydroxide (NaOH, 1N, Certified, Fisher Scientific, Waltham, MA, USA), sodium hypochlorite (NaClO, 11.9%, reagent grade, Sigma-Aldrich, Burlington, MA, USA), 2,2,6,6-tetramethylpy-peridine-1-oxyl (TEMPO, 99.9%, Sigma-Aldrich), sodium bromide (NaBr, BioXtra, 99.6%, Sigma-Aldrich), sodium periodate (NaIO_4_, 99.9%, Sigma-Aldrich), sodium chlorite (NaClO_2_, 80%, Sigma-Aldrich), glacial acetic acid (CH_3_COOH, 99.9%, Fisher Scientific), sodium hydroxide (NaOH, 1 N, Certified, Fisher Scientific), and deuterium oxide (D_2_O, 99.9%, Cambridge Isotope Laboratories, Inc., Tewksbury, MA, USA) were used as received without further purification. All water used was purified by a Milli-Q plus water purification system (Millipore Corporate, Billerica, MA, USA). The United States Department of Agriculture (USDA) Forest Product Laboratory (Madison, WI, USA) provided a dissolving soft wood pulp sheet.

### 2.2. Cellulose Nanofibrils by TEMPO-Mediated Oxidation (T-CNF)

The dissolving soft wood pulp sheet (1.0 g) was cut into 2 mm × 2 mm pieces, suspended in 100 mL water, and mixed with a magnetic stirrer for 2 h. An aqueous mixture (2 mL) containing 0.016 g TEMPO and 0.1 g NaBr was added into the aqueous cellulose suspension and stirred for 5 min. Oxidation reaction was initiated by adding an ca. 13.5 *w*/*v*% NaClO solution drop-wise to reach specific NaClO concentrations of 1.5, 3, 5, and 8 mmol per gram of cellulose at an ambient temperature. As oxidation proceeded, the decreased pH was adjusted to 10 ± 0.2 with 0.5 M of NaOH. The oxidation reaction ended when no acid was produced or the pH ceased to lower, lasting 30 and 50 min for the respective reactions with 1.5 and 3 mmol/g of NaClO_2_. For reactions with 5 and 8 mmol/g of NaClO, the respective reaction times were 60 and 80 min when the decreasing pH slowed to −0.03 pH per minute. All dispersions were neutralized to pH 7 by adding 0.5 M of HCl. Each suspension was centrifuged (5k rpm, 15 min) to decant the clear supernatant to be dialyzed (MWCO = 12–14 kDa) against purified water until the conductivity reached below 0.8 µs, typically in 3 d. The dialyzed suspensions (ca. 100 mL) appeared to be white turbid and were designated as T-Cell1.5, T-Cell3, T-Cell5, and T-Cell8 for the TEMPO-oxidized cellulose fibers with 1.5, 3, 5, and 8 mmol of NaClO_2_/g of cellulose, respectively. 

Each aqueous T-Cell suspension (0.1 *w*/*v*%, 100 mL) was transferred to a 2 L beaker and blended at 37k rpm (Vitamix 5200) for varying lengths of time; i.e., 1 to 60 min for T-Cell1.5, T-Cell3, and T-Cell5; 1 to 30 min for T-Cell5 and T-Cell8. The blended suspensions were allowed to settle at 4 °C for 24 h, centrifuged (Thermo Fisher Scientific; Megafuge 1.6 L) at 5k rpm for 15 min, then vacuum filtered (Whatman 541) to collect the CNF-containing supernatant. The TEMPO-oxidized CNF products were designated as T-CNFx.y where x denoted the NaClO concentration in mmol/g of cellulose and y was for the blending time in min; e.g., T-CNF3.30 for that from 3 mmol/g of NaClO and 30 min of blending. The yields of the CNFs were calculated based on the CNF mass in the supernatant over the TEMPO-oxidized cellulose (T-Cell) and reported in *w*/*v*%. 

### 2.3. Cellulose Nanofibrils by Periodate–Chlorite Oxidation (PC-CNF)

The dissolving soft wood pulp sheet (0.5 g, 3.08 mmol anhydroglucose unit or AGU) was cut into 2 mm × 2 mm pieces, suspended in 100 mL of water, and mixed with a magnetic stirrer. Primary oxidation with sodium periodate was conducted by adding NaIO_4_ (0.33 g, 1.54 mmol) at 0.5:1 NaIO_4_/AGU molar ratios in the dark at 55 °C (in an oil bath) for 4 h. The never-dried periodate-oxidized cellulose (P-cell) was dialyzed against water until its conductivity lowered to <10 μS cm^−1^, then concentrated to 100 mL via rotatory evaporation. A secondary sodium chlorite oxidation was proceeded by adding NaClO_2_ (0.14 g, 1.54 mmol) and CH_3_COOH (1.5 g. 0.5 M) to 50 mL of an aqueous P-cell suspension at a 1:1 NaClO_2_/AGU molar ratio at an ambient temperature for 0.5–24 h to produce periodate-chlorite oxidized cellulose (PC-cell). The never-dried PC-cell was dialyzed until its conductivity lowered to <1 μS cm^−1^. The aqueous PC-cell suspension was diluted to 200 mL and deprotonated with 0.5 M of NaOH (pH between 7–8) to convert surface carboxylic acid groups to charged sodium carboxylate. After blending (Vitamix 5200) at 37k rpm for 30 min and centrifugation (Thermo Fisher Scientific, Megafuge 1.6 L) at 5k rpm for 15 min, the aqueous supernatant was collected as periodate-chlorite oxidized nanofibrils (PC-CNFs). The PC-CNFs were denoted via their primary periodate oxidation molar ratio and secondary chlorite reaction time, e.g., PC-CNF0.5–12 h for that from NaIO_4_/AGU and 12 h of NaClO_2_ secondary oxidation.

### 2.4. Charge by Conductivity Titration 

HCl (100 µL, 1 N) was added to 50 mL of a 0.1 wt% T-CNF suspension or PC-CNF dispersion to protonate all the carboxyl groups, then titrated with 0.01 M of a NaOH solution. The conductivity values were recorded using an OAKTON pH/Con 510 series meter. The surface charge (σ, mmol/g of cellulose) was determined from the equation

(1) $$\sigma =\frac{c(v_{2}-v_{1})}{m}$$

where *c* is the NaOH concentration (M), m is the T-CNF or PC-CNF mass (g) in the suspension or dispersion, and $v_{1}$ and $v_{2}$ are the NaOH volumes (mL) used from neutralizing the added HCl and carboxylic acid on the T-CNFs or PC-CNFs, respectively. 

### 2.5. Dimensions by Imaging 

The thickness and length dimensions of the CNFs were determined using an atomic force microscope (AFM), whereas their widths were measured using a transmission electron microscope (TEM). T-CNFs (10 μL, 0.0002 *w*/*v*%) and PC-CNFs (10 μL, 0.0001 *w*/*v*%) were deposited on freshly cleaved hydrophilic mica, then air-dried in a fume hood for 1 h and profiled by an AFM using Asylum-Research MFP-3D in the tapping mode with OMCL-AC160TS standard silicon probes with a nominal tip radius of 7 nm and a 26 N/m force constant, 5 µm × 5 µm scan size, and 512 Hz rate. AFM thickness measurements were made across the widths of 100 nanofibrils from at least four images. T-CNFs (5 μL, 0.0002 *w*/*v*%) and PC-CNFs (10 μL, 0.0001 *w*/*v*%) were deposited on glow-discharged carbon-coated TEM grids, and excess liquid was removed after 15 min by blotting with a filter paper. To enhance image contrast and quality, the specimens were negatively stained with aqueous uranyl acetate (2 *w*/*v*%) and blotted to remove excess solution with filter paper, staining and blotting were repeated five times then dried under the ambient condition for 15 min. The samples were observed under a 200 kV acceleration voltage using an FEI Talos 120C TEM. The thickness (N = 100), width (N = 50), and length (N = 30 for AFM, N = 50 for TEM) of the CNFs were measured using ImageJ Analyzer (Java 1.8.0, NIH, USA) software with 0.7 nm/pixel for the image in a 100 nm scale bar. The width of each nanofibril at three different locations along the length were measured and the medium was used. The mean and standard deviation were calculated from samples collected from at least 4 images for each condition. 

### 2.6. Structural Characterization

For solution state ^1^H NMR characterization, aqueous CNF was solvent transferred from H_2_O to D_2_O via acetone. Acetone (40 mL) was added into the aqueous CNF dispersion (10 mL, 0.50 *w*/*v*%) followed by centrifugation (5k rpm, 10 min) to decant the supernatant. This acetone addition and centrifugation process was repeated three times to prepare a CNF acetone gel precipitate. The TEMPO-CNF acetone gel (ca. 5 mg) was added into 1 mL of D_2_O, then sonicated (10 min, Branson 2510) and vacuum evaporated at 50 °C for 1 h. This sonication–evaporation process was repeated three times to remove residual acetone. The transparent D_2_O suspension was transferred to an NMR tube for ^1^H NMR characterization on a Bruker AVIII 800 MHz NMR spectrometer. The crystalline structures were determined by X-ray diffraction (XRD) using a PANalytical X’pert Pro powder diffractometer with a Ni-filtered Cu Kα radiation (λ = 1.5406 Å) at a 45 kV anode voltage and 40 mA current. Aqueous T-CNF and PC-CNF (20 mL, 0.1 *w*/*v*%) dispersions were oven-dried (55 °C) overnight for the preparation of their respective films. Each sample was fixed on stage using double-sided tape, then a diffractogram was recorded from 5 to 40° at a scan rate of 2°/min. The crystallinity index (CrI) was calculated using the intensity of the 200 peak (I_200_, 2θ = 22.5°) and the intensity minimum between the peaks at 200 and 110 (I_am_, 2θ = 19.0°) as follows [22]:

(2) $$CrI=\frac{I_{200}-I_{am}}{I_{200}}$$

The surface primary C6 hydroxyl converted to carboxyl (mol per mol, OH/AGU) was determined as [23]:

(3) $$\mathrm{Surface}\;\mathrm{primary}\;\mathrm{hydroxyls}=\frac{\left(D/0.61+D/0.53\right)}{\left(D/0.61+1)\times (D/0.53+1\right)}$$

where 0.61 nm, 0.53 nm, and D are the d-spacings and average crystallite dimension of the 1$\bar{1}$0 and 110 crystallographic planes of cellulose I structures, respectively.

## 3. Results and Discussion

### 3.1. T-CNF from TEMPO-Mediated Oxidation and Blending

**Figure 1:**
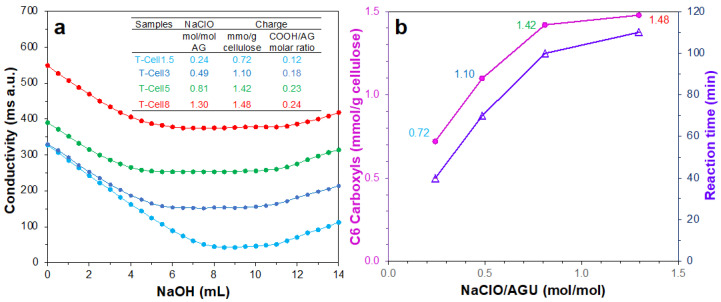
TEMPO-oxidized cellulose (T-Cell) synthesized at 1.5, 3, 5, and 8 mmol of NaClO/g of cellulose: (**a**) conductometric titration; (**b**) surface charges and reaction times.

The TEMPO-mediated C6 regioselective oxidation of the dissolving pulp was conducted at 1.5, 3, 5, and 8 mmol of NaClO per gram of cellulose to produce TEMPO-oxidized cellulose (T-Cell) series that were designated as T-Cell1.5, T-Cell3, T-Cell5, and T-Cell8, respectively (Figure 1a). These TEMPO oxidation reactions correspond to their respective 0.24, 0.48, 0.81, and 1.30 NaClO/anhydroglucose (AGU) ratios. The conversion of cellulose C6 hydroxyls to carboxyl groups was quantified via conductivity titration (Equation (1)) to show the surface charge of the T-Cell increased from 0.72 to 1.42 mmol/g with an increasing NaClO/AGU ratio from 0.241 to 0.81, then slightly increased to 1.48 mmol/g at 1.30 NaClO/AGU (Figure 1b). The length of time taken to complete the TEMPO oxidation reaction also exhibited the same trend, i.e., it increased from 40 to 100 min with an increasing NaClO/AGU ratio from 0.24 to 0.81, then a much smaller incline in time to 110 min with a considerable increase to 1.3 NaClO/AGU (Figure 1b). Thus, the TEMPO oxidation of pulp cellulose at 5 mmol of NaClO per gram of cellulose, corresponding to a 0.81 NaClO/AGU molar ratio, was deemed optimal to convert the most surface C6 hydroxyls (23% conversion) to carboxyl functional groups. 

**Figure 2:**
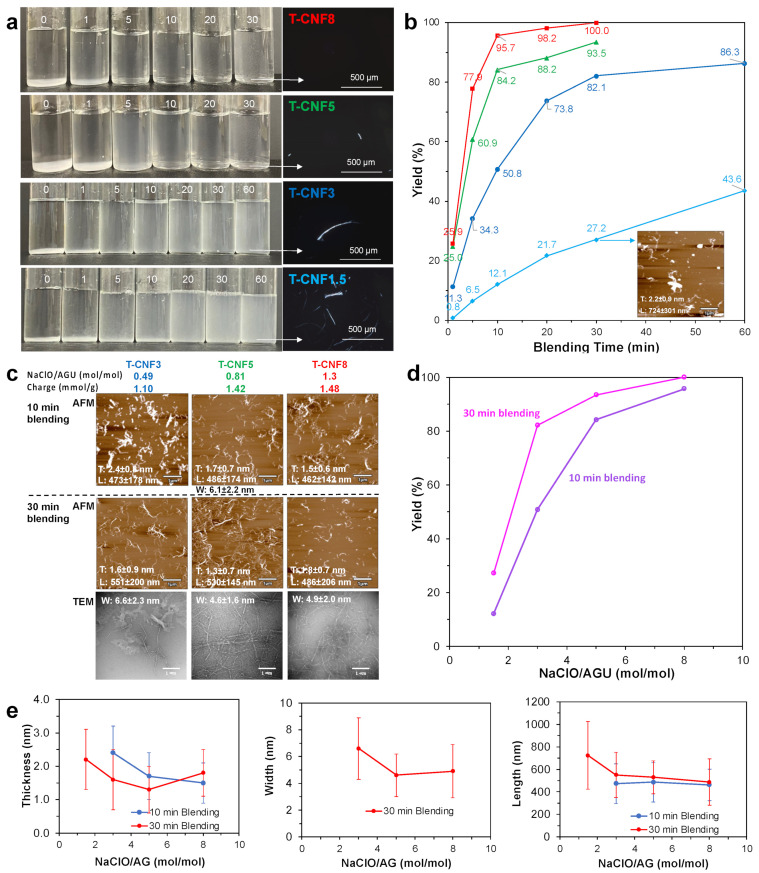
T-CNF from the TEMPO oxidation of dissolving pulp cellulose blended for varying lengths of time (min): (**a**) photographs of centrifuged 0.1% suspensions with optical microscopic images of precipitates under cross polarizers; (**b**) yields vs. blending time; (**c**) AFM and TEM images from blending for 10 and 30 min with their corresponding thickness (T), length (L), and width (W) dimensions and yields; (**d**) T-CNF yields; (**e**) T-CNF thickness, length, and width with their respective NaClO/AGU molar ratios. All AFM images have the same −2.5 to +2.5 nm z-scale bar as in Figure S1.

The four TEMPO-oxidized celluloses (T-Cell) with varied surface charges from 0.72 to 1.42 mmol/g of surface carboxyls were high-speed blended at a 0.1 *w*/*v*% concentration for varying lengths of time from 1 to 60 min, then centrifuged (5k rpm, 15 min) to separate the CNF in the supernatants (Figure 2a, Figure S1). At all oxidation levels, substantial precipitates were observed from the shortest 1 min blending, indicative of an insufficient shearing to disintegrate any of the TEMPO-oxidized cellulose significantly. With a higher oxidation and longer blending, less precipitates were present, showing more TEMPO-oxidized cellulose being disintegrated to be dispersed in the supernatants. With an increasing oxidation, the supernatants of the blended suspensions at any given blending time also appeared to be increasingly opaque, indicating increasing CNF quantities in the supernatants. 

These observations are consistent with the expectation that more TEMPO-oxidized cellulose requires less shearing to be disintegrated into nanocelluloses due to a greater extent of reduced hydrogen bonding and an increased electrostatic repulsion force in the non-crystalline domains. However, the least oxidized T-Cell1.5 produced modestly increasing yields with longer blending, producing only 43.6% T-CNF1.5 in large bundles and partly fibrillated structures even from the longest 60 min blending (Figure 2b). The substantial bundles and merely 43.6% yield observed for T-Cell1.5 blended for a respective 30 and 60 min gave a clear indication that the 0.72 mmol/g charge produced from 0.24 NaClO/AGU was too low to be effectively fibrillated into T-CNF. T-Cell3 with a 1.10 mmol/g charge produced by TEMPO oxidation at 0.49 NaClO/AGU was deemed the threshold level for T-CNF production. From the more oxidized T-Cell3, T-Cell5, and T-Cell8, T-CNF production was much more strongly dependent upon the length of blending, producing 50.8% T-CNF3, 84.2% T-CNF5, and 95.7% T-CNF8 after only 10 min, then further increasing to a respective 82.1, 93.5, and 100% from a moderate blending time of 30 min, illustrating the synergistic effects of TEMPO oxidation and mechanical blending in facilitating the disintegration of TEMPO-oxidized cellulose into T-CNFs. 

For T-Cell3, T-Cell5, and T-Cell8, the short 10 min of blending produced T-CNFs in a reduced thickness from 2.4 to 1.5 nm but in similar lengths between 462 to 486 nm (Figure 2c). Lengthening the blending to 30 min produced T-CNFs in an only slightly reduced thickness from 1.3 to 1.8 nm and lengths from 551 to 486 nm. In addition, widths of the T-CNFs also decreased from 6.6 nm to 4.6–4.9 nm when the surface charge increased from 1.10 mmol/g to 1.42–1.48 mmol/g. Overall, neither increasing the TEMPO oxidation level, i.e., the surface charges, nor the lengths of blending exhibited a clear trend in affecting T-CNF thickness, but T-CNF lengths seemed slightly shortened when the surface charge increased from 1.42 to 1.48 mmol/g, possibly by breaking the β-1,4-glycosidic link. Most significantly, all T-CNFs from the 30 min blending of T-Cell with 1.1 to 1.48 mmol/g charges were very homogeneous and in good to excellent yields of 82.1, 93.5, to 100%. Even 10 min of blending could produce 84.2 and 95.7% T-CNFs from the more charged 1.42 and 1.48 mmol/g T-Cell (Figure 2d).

Most significantly, the synergistic effects of TEMPO oxidation and mechanical blending are clearly evident and versatile to produce T-CNFs with surface charges ranging from 1.10 to 1.48 mmol/g but in similar dimensional attributes of a 1.3–2.4 nm thickness, 4.6–6.6 nm width, and 254–481 nm length (Figure 2e). Overall, T-Cell5 TEMPO oxidized at 0.81 NaClO/AGU molar ratios requires only 10 min of blending to produce an 84.2% T-CNF5 that could be maximized to 93.5% T-CNF5 with 30 min of blending. The even more oxidized T-Cell8 from a TEMPO oxidation at a 1.30 NaClO/AGU molar ratio could produce a 95.7% T-CNF8 with only 10 min blending or an impressive 100% could be converted with 30 min of blending. All T-CNFs are long-ribbon-like, i.e., highly dimensionally anisotropic with W/T ratios ranging from 2.7 to 4.1 and L/T ratios ranging from ca. 200 to 408 (Figure S1).

**Figure 3:**
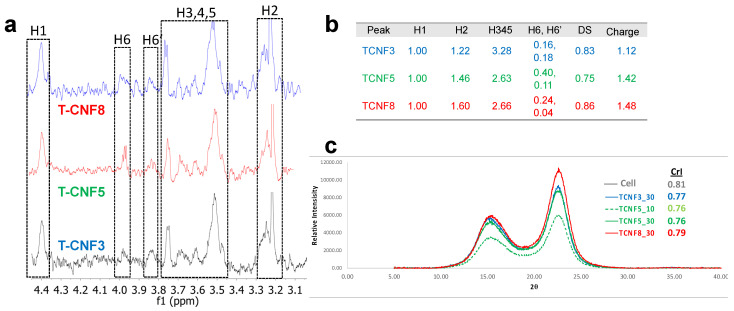
Characterizations of T-CNFs: (**a**) Solution state ^1^H NMR; (**b**) ^1^H peaks and DS values; (**c**) X-ray diffraction (XRD).

To further investigate the degree of substitution for T-CNFs, an ^1^H NMR was performed after the solvent changed from the aqueous dispersion to the D_2_O (Figure 3a). Under the assumption that all anomeric protons of amorphous and crystalline surface AGU of T-CNF are detectable by the ^1^H NMR, the degree of substitution (DS) of the T-CNF surface hydroxyls to carboxyls could be quantified. The cellulose anomeric proton was the sum of the integration of areas for all anomeric H1 to H5 proton peaks then averaged by five. Unmodified hydroxyls could be estimated by integrating the areas of H6 and H6′ then dividing by two protons. The DS of hydroxyls to carboxyls could be estimated by one minus the unmodified hydroxyl to anomeric proton ratio:

(4) $$DS=1-\frac{integral\;of\;(H6+H6^{\prime })/2}{\sum_{1}^{5}integral\;of\;{anomeric\;protons\;(H}^{i})/5}$$

The DS for T-CNF3 to T-CNF8 varied from 0.83, to 0.75, to 0.86, showing no clear association with surface charges, which may be due to the small portion of existing hydroxyls on the C6 (Figure 3b). The downfield peak at δ 4.4 was assigned to the cellulosic anomeric proton (H1) similar to the chemical shift at δ 4.5 reported in dissolved cellulose [6]. The H6 and H6′ peaks for T-CNF3-8 appeared at δ 3.97 and δ 3.85, comparable to the δ 3.65–3.88 range for dissolved MCC in NaOD/D_2_O [7]. Multiple overlapping peaks between δ 3.45–3.80 were assigned to H3, H4 and H5, matching those at δ 3.34–3.66 of the TEMPO-CNF [8]. The furthest up-field cellulosic peak at δ 3.2–3.3 coincided with the chemical shift of H2 at δ 3.2 for T-CNF in D_2_O [8]. 

The 0.81 CrI of dissolving pulp cellulose was reduced slightly to the 0.76 to 0.79 range for all T-CNFs via the coupled TEMPO oxidation and shear force from blending (Figure 3c). The indifferent CrI values indicate that the crystallinities of these T-CNFs are independent from the levels of TEMPO oxidation and blending time. The high yields as well as high and indifferent CrI are consistent with the relatively mild nature of TEMPO oxidation and the fact that all T-CNFs isolated in the supernatants were dialyzed and were thus free of lower molecular mass fragments. 

### 3.2. PC-CNF from Periodate–Chlorite Oxidation and Blending

**Figure 4:**
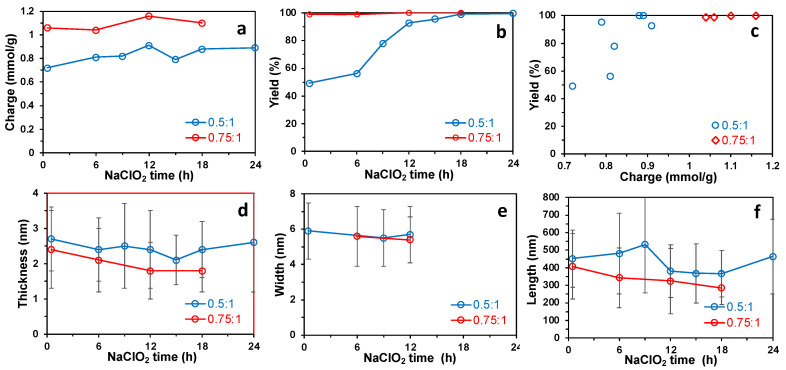
PC-CNFs synthesized from sequential periodate–chlorite oxidation at two primary NaIO_4_ oxidant levels of 0.5:1 and 0.75:1 NaIO_4_/AGU (55 °C, 4 h) and over a varying secondary NaClO_2_ (1:1 NaClO_2_/AGU) oxidation time followed by 30 min of blending: (**a**) charge; (**b**) yield; (**c**) yield vs. charge; (**d**) thickness measured via AFM; (**e**) width measured via TEM; (**f**) length measured via AFM.

The sequential periodate–chlorite oxidation of cellulose was conducted at two primary sodium periodate oxidant levels, i.e., 0.5:1 and 0.75:1 NaIO_4_:AGU molar ratios, at 55 °C for 4 h followed by secondary sodium chlorite oxidation at a 1:1 NaClO_2_:AGU molar ratio at an ambient temperature over varying lengths of time from 0.5 h to up to 24 h. Generally, a higher primary oxidant level led to higher charges and yields of PC-CNF (Figure 4a,b, Figure S2). With an increasing secondary NaClO_2_ oxidation time, the PC-CNF charges slightly increased from 0.72 to 0.89 mmol/g and from 1.06 to 1.16 mmol/g at 0.5:1 and 0.75:1 NaIO_4_:AGU molar ratios, respectively (Figure 4a). The PC-CNF yields, on the other hand, significantly increased from 49.3% to 99.6% with a longer secondary oxidation, with the most drastic increases occurring between 6 and 12 h at the lower primary oxidation level. In contrast, yields were impressively consistent at 100% and irrespective of the lengths of the reaction time at the higher primary oxidation level (Figure 4b). PC-CNF yields were linked with a higher charge, showing an over 90% yield when surface charges exceeded 0.88 mmol/g (Figure 4c). The PC-CNFs produced from the higher primary 0.75:1 NaIO_4_:AGU oxidant level were generally thinner and shorter but similar in widths to those from the lower primary 0.5:1 NaIO_4_:AGU oxidant level (Figure 4d–f). With a longer secondary oxidation, both the PC-CNF thickness and length reduced slightly, i.e., the thickness lowered from 2.7 to 2.1 nm and from 2.1 to 1.8 nm whereas the length shortened from 533 to 464 nm and from 407 to 285 nm at 0.5:1 and 0.75:1 NaIO_4_:AGU, respectively (Figure 4d). The widths of all PC-CNFs were in between 5.4 and 5.9 nm, seemingly independent of either the primary NaIO_4_ levels or the lengths of secondary oxidation (Figure 4e). All PC-CNFs are dimensionally anisotropic, W/T and L/T ratios exceed 1 and 100, respectively.

**Figure 5:**
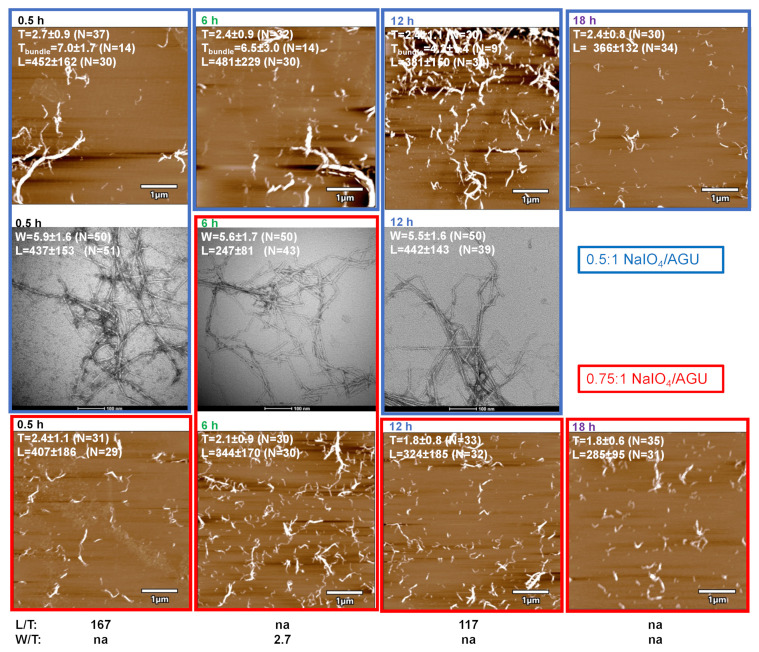
AFM (top and bottom row) and TEM (middle row) images of PC-CNF from sequential periodate–chlorite oxidation (55 °C, 4 h) at two primary NaIO_4_ oxidant levels of 0.5:1 (blue box) and 0.75:1 (red box) NaIO_4_/AGU (55 °C, 4 h) and varying secondary NaClO_2_ (1:1 NaClO_2_/AGU) oxidation times followed by 30 min of blending.

The quality of the PC-CNFs was further elucidated by imaging the diluted supernatants. Substantial bundles observed from the lower 0.5:1 NaIO_4_/AGU primary oxidation and shorter secondary oxidation up to 6 h gave clear evidence of the partial defibrillation of the 56.3% in the supernatant (Figure 5). Even with the much improved 92.8% yield from the longer 12 h of secondary oxidation, some bundled structures were still observed by both AFM and TEM. The bundle thickness reduced from 7.0 nm to 4.2 nm as the secondary oxidation lengthened from 0.5 h to 12 h, then no fiber bundle was observed at 18 h. From the lower primary oxidation at 0.5:1 NaIO_4_/AGU, the relative uniform PC-CNF (T: 2.1 ± 0.7 nm) in a high yield (95.5%) required longer secondary oxidation at 15 h (Figure S2). On the contrary, the higher primary oxidation at 0.75:1 NaIO_4_/AGU produced exclusively isolated PC-CNFs, irrespective of the lengths of the secondary oxidation and, impressively, from as brief as only 0.5 h. However, a longer secondary chlorite oxidation did reduce PC-CNF dimensions as noted earlier (Figure 4), indicative of β-1,4-glycosidic link chain scission. All PC-CNFs are dimensionally anisotropic. The W/T ratio was 2.7 and L/T ratios ranged from 117 to 167 among the few with corresponding T, W, and L values. 

**Figure 6:**
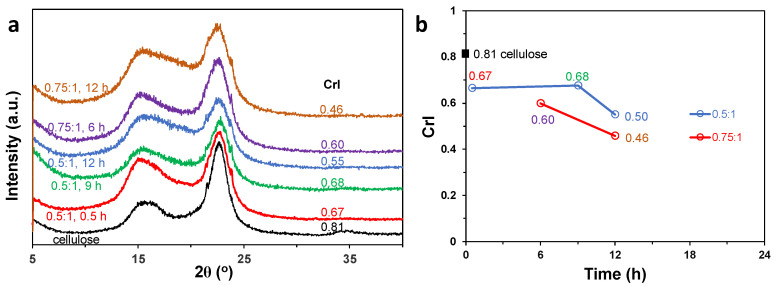
PC-CNF characterization: (**a**) XRD spectra; (**b**) crystallinity index (CrI) over varying secondary oxidation times.

The crystallinity of all the PC-CNFs were significantly lower than the 0.81 CrI of the original dissolving pulp cellulose (Figure 6a,b). From the primary oxidation at the lower 0.5:1 NaIO_4_/AGU ratio, the CrI was significantly reduced to 0.67 and remained there with increasing lengths of secondary oxidation time from 0.5 to 9 h, then lowered further to 0.55 at 12 h. From the primary oxidation at the higher 0.75:1 NaIO_4_/AGU molar ratio, the CrI was also significantly reduced to 0.60 at 6 h then lowered even further to 0.46 at 9 h of secondary oxidation time. That most significant CrI reduction was observed with even a brief secondary oxidation supports the notion that breaking the C2-C3 bond in the primary periodate oxidation imposes the most significant effect on reducing the crystallinity, and periodate oxidation occurs not only on the amorphous chains but also across the amorphous-crystalline boundaries into the crystalline domains to reduce crystallinity. A longer secondary chlorite oxidation of C2,C3 dialdehydes into dicarboxls further reduces the crystallinity. 

### 3.3. Regioselective C6 vs. C2,C3 Carboxylation Comparison

Both TEMPO-mediated and sequential periodate–chlorite oxidation reactions involve the heterogeneous oxidation of respective C6 and C2,C3 hydroxyls in the less-ordered and the amorphous-crystalline interfacial regions of the micro-scale cellulose. In the TEMPO-mediated oxidation involving NaBr and NaClO, both TEMPO and NaBr serve as catalysts and NaClO as the primary oxidant. NaClO oxidizes NaBr into the much stronger secondary hypobromite NaBrO oxidant that oxidizes TEMPO to the N-oxoammonium ion to convert cellulose C6 hydroxyls to aldehydes. The C6 aldehyde-to-carboxyl conversion requires another equivalent N-oxoammonium ion. The N-oxoammonium ions reduce to hydroxylamine then are oxidized back to TEMPO nitroxyl radicals to repeat the cycle. Therefore, each C6 hydroxyl-to-carboxyl oxidation requires two molar equivalents of TEMPO/NaBr/NaClO. With a 0.81 CrI of the dissolving pulp cellulose, the threshold 0.49 NaClO/AGU oxidant level that yielded 82.1% T-CNF3.30 is slightly higher than two times that of the 0.19 molar equivalent of the non-crystalline cellulose fraction and is stoichiometrically consistent when taking the amorphous-crystalline interfacial areas into account. Above the threshold, TEMPO oxidized at 0.81 NaClO/AGU carrying a 1.4 mmol/g charge produced 84.2% T-CNF with only 10 min of blending or 93.5% T-CNF with 30 min of blending. A higher TEMPO oxidation at 1.3 NaClO/AGU could produce 95.7% T-CNF with only 10 min of blending or an impressive 100% converted with 30 min of blending.

In a sequential periodate–chlorite oxidation, sodium periodate breaks the C2-C3 bonds in the anhydroglucose ring in the primary oxidation to form C2,C3 dialdehydes that are subsequently oxidized by sodium chlorite into dicarboxylates in the secondary oxidation. The 0.5 primary oxidation NaIO_4_/AGU ratio, i.e., over two times that of the 0.19 molar equivalent of the non-crystalline cellulose fraction, was deemed necessary as the primary at 0.25 NaIO_4_/AGU was insufficient for the secondary oxidation to proceed. Such a significant excess of the primary NaIO_4_ oxidant suggests its lower accessibility to the C2-C3 bonds than the N-oxoammonium ion to the primary C6 hydroxyls in the case of the TEMPO.

The TEMPO oxidation is a milder oxidation reaction at an ambient temperature and more time efficient due to the built-in sequential oxidation mechanism in its one-pot reaction, whereas the periodate–chlorite oxidation involves a two-step oxidation which is required to be conducted in the dark and at a moderately elevated 55 °C temperature. The T-CNFs produced also possess a wider range of 0.72–1.48 mmol/g charges on C6 compared to the narrower charge range of 0.72–1.16 mmol/g on the C2 and C3 of PC-CNF. 

The optimally synthesized T-CNFs were averaged to be 1.3 nm thick, 4.6 nm wide, and 254 nm long in a 93.5% yield from a TEMPO oxidation at a 0.81:1 NaClO:AGU molar ratio and 30 min of blending. The optimally produced PC-CNFs were 2.4 nm thick, 5.5 nm wide, and 442 nm long on average in the 92.8% yield from the primary sodium periodate oxidation at a 0.5:1 NaIO_4_:AGU molar ratio and 12 h of secondary sodium chlorite oxidation at a 1:1 NaIO_4_:AGU molar ratio and 30 min of blending. Both T-CNFs and PC-CNFs are long-ribbon-like, i.e., highly dimensionally anisotropic with W/T ratios ranging from 2.7 to 4.1 for T-CNFs and 2.7 for one PC-CNF and with L/T ratios ranging from ca. 200 to 408 for T-CNFs and 117–167 for two PC-CNFs. Both regioselective oxidizations could be easily tuned by optimizing the respective oxidant to the accessible AGU in the dissolving pulp to convert over 90% of the cellulose into T-CNFs and PC-CNFs. The minimal charge levels for efficient shear force blending into CNF were slightly higher for the T-Cell at 1.1 mmol/g than the PC-Cell’s 0.88 mmol/g. The dimensional attributes of both T-CNFs and PC-CNFs fall within similar ranges of thicknesses (1.3–2.4 nm T-CNF, 1.8–2.7 nm PC-CNF), widths (4.6–6.6 nm T-CNF, 5.5–5.9 nm PC-CNF), and lengths (254–481 nm T-CNF, 247–442 nm PC-CNF).

TEMPO-mediated oxidation is an energy- and time-efficient way to prepare slightly thinner and shorter C6 carboxylated CNFs; sequential periodate–chlorite oxidation is versatile to produce a slightly longer C2,C3 dicarboxylated CNF in which the dialdehyde intermediates are also amenable to further chemical reactions to generate multiple types of functionalized nanocelluloses as well as other reactive nanomaterials. 

Though both TEMPO and PC oxidation reactions have been reported individually on various cellulose sources, these regioselectively oxidized and well-characterized C6 and C2,C3 carboxylated CNFs from unmodified cellulose represent the first comparable series of nanocellulose. Generated from mass-produced, commercially available, and relatively pure dissolving pulp, these CNFs represent the first comparable series of regioselectively carboxylated nanocellulose references and standards. Specifically, these regioselectively carboxylated CNF series are presented as a range of well-characterized C6 and C2,C3 carboxylated CNFs along with C6 sulfated CNFs [19] for the development of a toolbox of safer-by-design substance evaluation tools linking the biological behavior of functionalized CNFs to their surface chemistries, charges, and dimensions [20]. These approaches may be further expanded to apply to less-purified or other sources of cellulose as well as to advance future scalable and industrially relevant product and application development.

## 4. Conclusions

Regioselective oxidation has been stoichiometrically applied to dissolving pulp cellulose and synergistically coupled with high-speed blending (30k rpm, 10–30 min) to optimize the production of C6 and C2,C3 carboxylated CNFs via a respective one-pot TEMPO-mediated and sequential sodium periodate (NaIO_4_)/sodium chlorite (NaClO_2_) (PC) oxidation with robust yields and varied CNF charge and dimensional attributes. The levels of both oxidation reactions were easily tuned stoichiometrically by varying the oxidant to cellulose anydroglucose unit (AGU) molar ratios and the lengths of the reaction time. TEMPO-mediated oxidation at 0.24, 0.49, 0.81, and 1.30 NaClO/AGU led to surface charges ranging from 0.72 to 1.42 mmol/g. TEMPO oxidation at 0.49 NaClO/AGU produced a 1.10 mmol/g charge that was 93.5% disintegrated into T-CNFs. The 0.49 NaClO/AGU threshold oxidant level was stoichiometrically consistent, considering the slight excess over two times the equivalent of the 0.19 non-crystalline fraction to access chains in the amorphous regions and amorphous-crystalline interfaces. The sequential periodate–chlorite oxidation of cellulose conducted at a stoichiometrically excess primary periodate oxidation, i.e., 0.5:1 and 0.75:1 NaIO_4_:AGU levels, and varying the secondary chlorite oxidation time from 0.5 to 24 h produced C2,C3 carboxylated PC-CNF with charges ranging from 0.72 to 1.16 mmol/g. Generally, higher primary oxidant levels led to higher charges and yields of PC-CNF, and an over 90% yield was typical when surface charges exceeded 0.88 mmol/g. Both CNF series have similar ranges of dimensions, i.e., thicknesses (1.3–2.4 nm for T-CNF, 1.8–2.7 nm PC-CNF), widths (4.6–6.6 nm T-CNF, 5.5–5.9 nm PC-CNF), and lengths (254–481 nm T-CNF, 247–442 nm PC-CNF).

Both oxidation approaches are robust, capable of producing CNFs at high yields of over 90% to nearly 100%. From the perspective of efficiency, the one-pot TEMPO-mediated oxidation is milder and quicker to produce C6 carboxylated T-CNF whereas the sequential periodate–chlorite oxidation produces slightly longer C2,C3 dicarboxylated CP-CNF in which the dialdehyde intermediate is reactive and useful for further functionalization toward creating other chemically functionalized CNFs. These systematically oxidized and well-characterized C6 and C2,C3 carboxylated CNFs from relatively pure and industrially available dissolving pulp cellulose represent the first comparable series of critically needed nanocellulose references and standards and are foundational to future nanocellulose product and application development.

## Data Availability

The original contributions presented in the study are included in the article/Supplementary Materials, further inquiries can be directed to the corresponding author.

## Supplementary Materials

The following supporting information can be downloaded at: https://www.mdpi.com/article/10.3390/nano14050479/s1, Figure S1: Yields and charge of T-CNFs from blending (5k rpm) of (a) Cell3 (10 min), (b) Cell3 (30 min), (c) Cell5 (10 min), (d) Cell5 (30 min), (e) Cell8 (10 min), and (f) Cell8 (30 min). AFM were imaged on mica with corresponding thickness (N=100) and length (N=30) distribution at 0.0002 w/v%; Table S1: PC-CNF from sequential periodate-chlorite oxidation followed by blending (37,000 rpm, 30 min) at (a,d,c) 0.5:1 or (d,e,f) 0.75:1 NaIO4/AG primary NaIO4 oxidation (55 ºC, 4 h) and varying secondary NaClO2 (1:1 NaClO2/AG) oxidation time followed by 30 min blending: (a,d) yield and charge, (b,e) conductivity, (c,f) yield.
