# Supplementary material for: Regioselectively Carboxylated Cellulose Nanofibril Models from Dissolving Pulp: C6 via TEMPO Oxidation and C2,C3 via Periodate–Chlorite Oxidation
Source: Nanomaterials (Basel). 2024 Mar 6;14(5):479. doi: 10.3390/nano14050479 (PMC10934283; doi:10.3390/nano14050479)
Supplement: Supplementary file 1 [file nanomaterials-14-00479-s001.zip › nanomaterials-2890762-supplementary.pdf]

Supplementary Information

Regio-selective carboxylated cellulose nanofibrils from dissolving pulp: C6 via TEMPO-mediated oxidation and C2,C3 via periodate-chlorite oxidation

Mengzhe Guo[1], James Ede[2], Christie Sayes[3], Jo Anne Shatkin[2], Nicole Stark[4], You-Lo Hsieh[1]*

[1]Biological and Agricultural Engineering, Chemical Engineering, University of California at Davis, Davis, CA 95616-8722
[2]Vireo Advisors, LLC, PO Box 51368, Boston, MA, 02130
[3]Environmental Science, Baylor University, Waco, TX 76798-7266
[4]USDA Forest Service, Forest Products Laboratory, Madison, WI 53726-2398

*Email: ylhsieh@ucdavis.edu; Tel: +1 530 752 0843

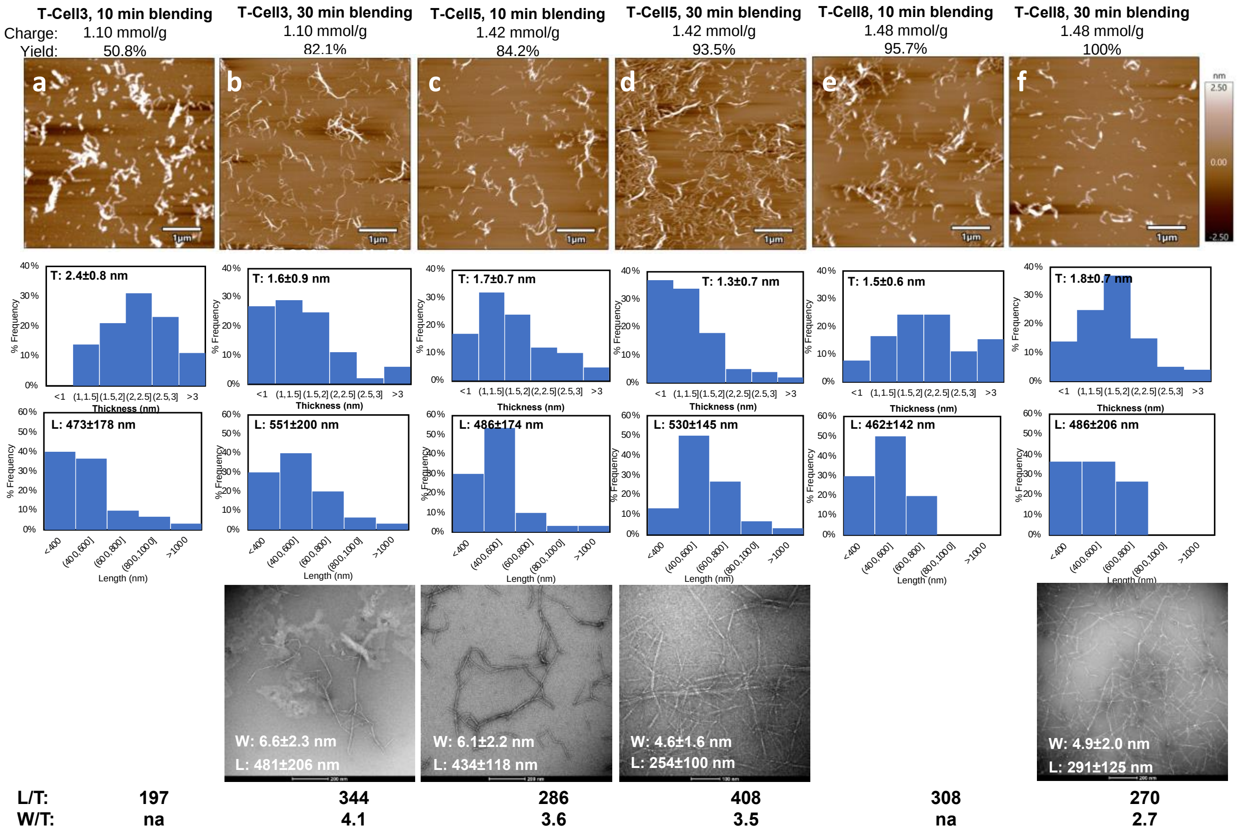

Figure S1. Yields and charge of T-CNFs from blending (5k rpm) of (a) Cell3 (10 min), (b) Cell3 (30 min), (c) Cell5 (10 min), (d) Cell5 (30 min), (e) Cell8 (10 min), and (f) Cell8 (30 min). AFM were imaged on mica with corresponding thickness (N=100) and length (N=30) distribution at 0.0002 w/v%.

1.1 Sodium Hypochlorite Titration

NaI (1.26 g, 8.4 mmol) was dissolved in acetic acid (4 mL) and mixture was diluted to 100 mL with purified water. NaI solution (100 mL) was divided into two equal 50 mL solutions with 1 mL NaClO solution (ca. 13.5 w/v%) added to each for dark red color solution. 0.1 M $Na_2S_2O_3$ solution was drop-wise (0.1 mL) added into NaI solution until red color faded and total volume was recorded.

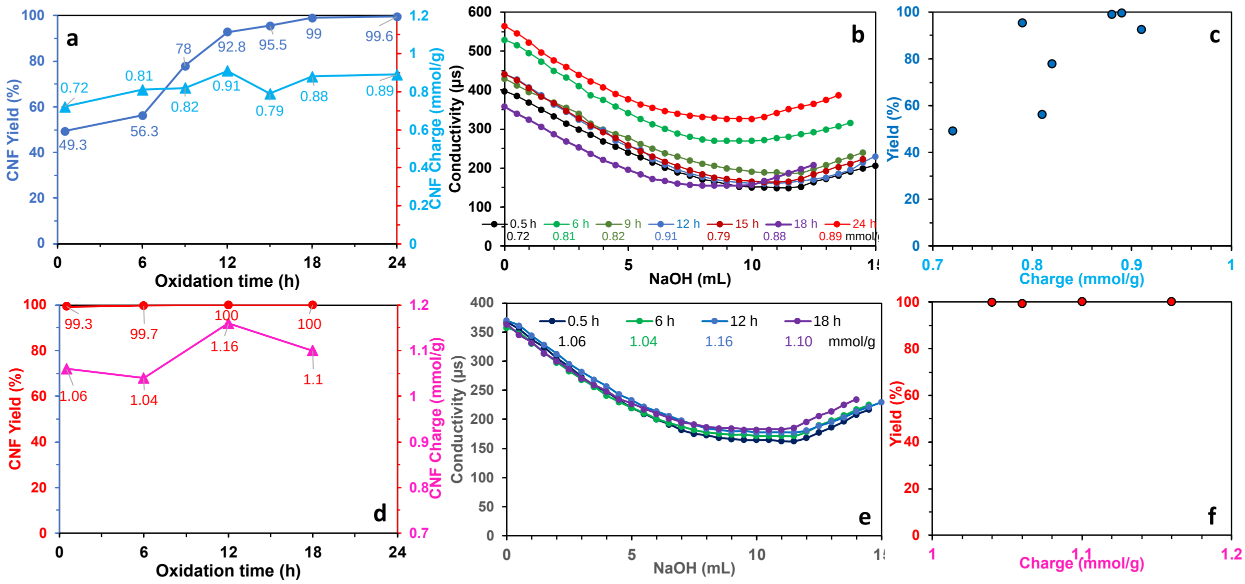

Figure S2. PC-CNF from sequential periodate-chlorite oxidation followed by blending (37,000 rpm, 30 min) at (a,d,c) 0.5:1 or (d,e,f) 0.75:1 $NaIO_4$/AG primary $NaIO_4$ oxidation (55 ºC, 4 h) and varying secondary $NaClO_2$ (1:1 $NaClO_2$/AG) oxidation time followed by 30 min blending: (a,d) yield and charge, (b,e) conductivity, (c,f) yield.
